# Medicinal Plant-Based Naturopathy and Its Therapeutic Potential: A Systematic Review of Clinical and Life Science Evidence

**DOI:** 10.7759/cureus.110225

**Published:** 2026-06-04

**Authors:** Gokula Krishnan B, Deepti Sharma, Manisha Kumari, Reet Arora, Km Shweta Malik

**Affiliations:** 1 Department of Naturopathy and Yoga, Maharishi Aurobindo Subharti College and Hospital of Naturopathy and Yogic Sciences, Swami Vivekanand Subharti University, Meerut, IND

**Keywords:** adaptogens, complementary and alternative medicine, ethnobotany, medicinal plants, naturopathy, phytotherapy

## Abstract

Medicinal plants have been used in traditional healthcare practices and remain a significant source of therapeutic agents, largely due to diverse bioactive phytochemicals with pharmacological properties relevant to disease prevention and management. Scientific interest in medicinal plant-based naturopathy has increased in recent years, prompting systematic investigations through clinical and life science research to evaluate therapeutic effectiveness and underlying mechanisms. This systematic review aimed to assess the therapeutic potential of medicinal plant-based naturopathy by examining clinical and experimental evidence related to plant-derived interventions for various health conditions. A structured literature search was conducted across major databases, including PubMed, Scopus, Web of Science, and Google Scholar, to identify studies published between 2014 and 2026, and studies were screened based on predefined inclusion criteria focusing on medicinal plant interventions. Eligible studies included randomised controlled trials, placebo-controlled clinical trials, and supporting life science research, with data extracted and analysed qualitatively to summarise study characteristics, therapeutic outcomes, and methodological aspects. Methodological quality and potential bias were assessed using the Cochrane risk-of-bias framework, with results visualised using the ROBviz tool. This systematic review was prospectively registered in the International Prospective Register of Systematic Reviews (PROSPERO) under registration number CRD420261333889. Evidence from the included studies indicates that several medicinal plants demonstrate potential in managing metabolic disorders, gastrointestinal diseases, musculoskeletal pain, reproductive health conditions, and psychological stress, primarily through antioxidant, anti-inflammatory, antimicrobial, and adaptogenic mechanisms. Further large-scale clinical studies are required to strengthen the evidence base.

## Introduction and background

Medicinal plants have long been utilised in traditional healthcare systems and are important sources of bioactive compounds with therapeutic properties for preventing and managing diseases [1]. In this review, medicinal plant-based naturopathy refers to the use of whole plants, plant extracts, plant-derived compounds, or traditional plant-based formulations as supportive or complementary approaches to health management. Interest in these therapies has increased because plant-derived interventions may offer therapeutic effects with comparatively fewer adverse effects than many synthetic drugs [2]. Recent biomedical research has also shown that medicinal plant-derived nanovesicles may act as regulatory mediators in cellular microenvironments, suggesting additional mechanisms by which plant-based therapies may influence disease processes [3]. Medicinal plants have been investigated in chronic diseases, such as chronic kidney disease, type 2 diabetes mellitus, hypertension, dyslipidemia, non-alcoholic fatty liver disease, Alzheimer’s disease, Parkinson’s disease, depression, respiratory disorders, and cancer. Their bioactive compounds may help reduce oxidative stress and inflammation, including in chronic kidney disease and related complications [4]. Advances in phytochemical and analytical methods have further improved the identification and characterisation of active compounds responsible for these therapeutic effects [5].

Metabolic and neurological conditions represent two major areas of medicinal plant research. In metabolic disorders, medicinal plants have been studied for diabetes mellitus because some plant-derived compounds may modulate blood glucose levels and improve insulin sensitivity [6]. In neurological disorders, traditional medical systems, such as Ayurveda, have used plant-based formulations to support the management of Parkinson’s disease through possible neuroprotective effects [7]. Alzheimer’s disease has also been investigated in relation to medicinal plant-based therapies, as several herbs contain antioxidant, anti-inflammatory, and neuroprotective compounds that may help limit neuronal damage and cognitive decline [8]. Medicinal plants have also been investigated for immune-related and antiviral applications, particularly during the coronavirus disease 2019 pandemic, where they were considered as potential supportive agents because of their antiviral and immunomodulatory properties [9]. At the same time, modern biotechnology, including gene editing, has strengthened medicinal plant research by improving plant traits and phytochemical production for drug discovery [10]. Additional life science evidence has explored the neuroprotective activity of natural compounds from medicinal plants [11], while experimental models such as Caenorhabditis elegans and zebrafish are increasingly used to study anti-ageing and neurological effects of plant-derived compounds [12,13].

In infectious diseases, medicinal plants are known for their antimicrobial properties and have been studied for infectious disease control [14]. Examples include* Allium sativum*, which contains allicin and is associated with antibacterial activity; *Azadirachta indica*, which contains nimbidin and azadirachtin and has been used in relation to skin, oral, and wound-related infections; *Curcuma longa*, which contains curcumin and has antibacterial and antifungal potential; and *Ocimum tenuiflorum*, which contains eugenol and ursolic acid and has been studied for respiratory and microbial infections. These examples indicate that plant-derived compounds may have antibacterial, antifungal, and antiviral effects relevant to both traditional medicine and modern pharmacological research [15].

Dermatological, hepatobiliary, and metabolic applications have also been described. In dermatological conditions, plant-based therapies have been investigated for inflammatory skin disorders and wound healing, where bioactive compounds may reduce inflammation and promote tissue repair [16]. In metabolic and liver diseases, medicinal plants have attracted interest because their compounds may influence lipid metabolism and hepatic function [17]. Reported laboratory parameters include liver function tests, such as alanine aminotransferase and aspartate aminotransferase, and lipid profile measures, including total cholesterol, triglycerides, low-density lipoprotein cholesterol, and high-density lipoprotein cholesterol [18]. Clinical and experimental studies also suggest possible roles for medicinal plants and natural compounds in liver disorders through antioxidant and anti-inflammatory mechanisms [19]. Their anti-inflammatory and antimicrobial properties further support their relevance in dermatological and dermatovenerological conditions [20].

Psychological and respiratory conditions form another important area of investigation. In mental health disorders, such as depression, several herbal compounds may affect neurotransmitter pathways and show antidepressant-like effects [21]. In respiratory diseases, medicinal plants have been studied for anti-inflammatory, antimicrobial, and immunomodulatory properties, which may be relevant where infection, inflammation, and immune dysregulation contribute to symptoms [22]. Ethnobotanical research continues to document traditional knowledge and identify plants used by local communities for disease management [23]. In cancer-related care, medicinal plants are used mainly as complementary approaches. Examples include *Curcuma longa* and curcumin in colorectal, breast, and pancreatic cancer research; *Camellia sinensis *and epigallocatechin gallate in prostate, breast, and colorectal cancer prevention research; *Nigella sativa* and thymoquinone in breast, colon, lung, and pancreatic cancer models; and *Catharanthus roseus*, which is a source of vinca alkaloids, such as vincristine and vinblastine, used in hematological malignancies, including leukemia and Hodgkin lymphoma [24]. Indigenous knowledge remains important for discovering new pharmacologically active compounds from medicinal plants [25].

Despite this broad therapeutic interest, the current evidence remains fragmented. Studies differ in plant species, active compounds, formulations, dosages, treatment durations, disease conditions, comparators, and outcome measures. Many reports focus on single plants or single disease areas, while fewer syntheses clearly connect clinical findings with supporting life science evidence. This creates uncertainty about which medicinal plant interventions have the strongest evidence, which therapeutic areas are most consistently supported, and which mechanisms are most relevant to observed clinical outcomes.

Objectives of the review

This systematic review aimed to assess the therapeutic potential of medicinal plant-based naturopathy by synthesising available clinical and life science evidence on plant-derived interventions. The review focused on evidence related to metabolic, gastrointestinal, musculoskeletal, reproductive, psychological, hepatic, infectious, respiratory, dermatological, neurological, and cancer-related conditions. It also considered therapeutic outcomes, mechanistic relevance, methodological quality, and remaining evidence gaps.

## Review

Methodology

A systematic review methodology was adopted to evaluate the therapeutic potential of medicinal plant-based naturopathy using clinical and life science evidence. The review was designed and reported in accordance with the Preferred Reporting Items for Systematic Reviews and Meta-Analyses (PRISMA) guidelines [26]. The methodology focused on identifying, selecting, appraising, and synthesising peer-reviewed studies that investigated medicinal plants as therapeutic interventions. Emphasis was placed on clinical relevance, methodological transparency, reproducibility, and evidence quality. The review aimed to provide a structured assessment of medicinal plant interventions used as supportive approaches in naturopathic healthcare practice.

Protocol Registration

The review protocol was prospectively registered in the International Prospective Register of Systematic Reviews (PROSPERO) under registration number CRD420261333889. The protocol was registered on March 20, 2026, and is available through the PROSPERO database. The registered protocol defined the review objectives, eligibility criteria, search strategy, screening process, data extraction plan, risk-of-bias assessment, and method of evidence synthesis before full study selection was completed. Protocol registration was undertaken to strengthen transparency, minimise reporting bias, and ensure that the review process followed a predefined methodological framework throughout the study.

Literature Search Strategy

A comprehensive literature search was conducted in PubMed, Scopus, Web of Science, and Google Scholar to identify clinical and life science evidence on medicinal plant-based naturopathic interventions published in English between 2014 and 2026. The search strategy combined three main concept groups using Boolean operators: medicinal plant terms, clinical study terms, and outcome-related terms. The core Boolean search string was: (“medicinal plants” OR “herbal medicine” OR “phytotherapy” OR “plant extracts” OR “naturopathy” OR “plant-based intervention”) AND (“randomized controlled trial” OR “randomised controlled trial” OR “controlled clinical trial” OR “placebo” OR “clinical trial” OR “human study”) AND (“efficacy” OR “safety” OR “therapeutic effects” OR “antioxidant” OR “anti-inflammatory” OR “antimicrobial” OR “adaptogenic”). In PubMed, equivalent MeSH and title/abstract terms were used. In Scopus, the search was adapted to TITLE-ABS-KEY fields, and in Web of Science, Topic Search was used. Google Scholar searches were performed using phrase-based combinations of the same search concepts. Supplementary plant-specific searches were performed using the names of plants and extracts identified in the eligible literature, including *Withania somnifera*, *Mentha piperita*, Tongxie Yaofang, *Silybum marianum*, *Morus alba*, *B. serrata*, *O. tenuiflorum*, menthol, limonene, and gingerol, combined with clinical-trial and outcome terms. Reference lists of relevant articles were manually screened to identify additional eligible studies.

Study Selection Criteria

Eligible studies included randomised controlled trials, placebo-controlled clinical trials, controlled clinical studies, and experimental human studies evaluating the therapeutic effects of medicinal plant-based interventions. Studies were required to report clearly defined health-related or physiological outcomes, intervention details, and sufficient data for qualitative interpretation. Studies published in English and available as full texts were considered for inclusion. Studies were excluded if they lacked clear outcome measures, involved non-therapeutic uses of plants, were not conducted in humans, were unavailable in English, or did not provide adequate methodological information for review assessment.

Screening and Eligibility Assessment

All records identified through database searching were imported into a reference management system, and duplicate records were removed before screening. Two reviewers independently screened the titles and abstracts of all retrieved records against the predefined inclusion and exclusion criteria. Records considered potentially eligible by either reviewer were advanced to full-text assessment. Full-text articles were then assessed against the predefined inclusion and exclusion criteria. Full-text eligibility assessment was also performed independently by the same two reviewers. Any disagreements regarding study inclusion were resolved through discussion, and a third reviewer was consulted when consensus could not be reached. Studies meeting all eligibility requirements were included in the final synthesis. Reasons for exclusion at the full-text stage were documented to maintain transparency. The study selection process was presented using a PRISMA flow diagram showing identification, screening, eligibility assessment, and final inclusion.

Data Extraction

Data were extracted independently by two reviewers using a structured data extraction form developed for the review. Extracted variables included author name, year of publication, country, medicinal plant investigated, study design, sample size, participant characteristics, intervention type, dosage, treatment duration, comparator group, outcome measures, and reported therapeutic effects. Data extraction focused on capturing information relevant to clinical efficacy, intervention characteristics, and health outcomes. Extracted information was compared between reviewers for consistency and completeness before synthesis, and discrepancies were resolved through discussion. Any unclear or incomplete information was recorded to avoid overinterpretation and to preserve accuracy in the presentation of evidence.

Risk-of-Bias Assessment

The methodological quality of included studies was assessed using the Cochrane Risk of Bias framework [27]. Assessment domains included random sequence generation, allocation concealment, blinding of participants and personnel, blinding of outcome assessment, completeness of outcome data, selective outcome reporting, and other potential sources of bias. Each domain was judged as low, high, or unclear risk of bias based on the information reported in each study. The results were visualised using the ROBviz tool. This assessment helped determine the reliability of included evidence and supported cautious interpretation of therapeutic claims.

Data Synthesis

The findings were synthesised qualitatively due to expected variation in medicinal plant species, intervention forms, dosage regimens, treatment durations, study populations, and reported outcomes. Therapeutic effects were grouped according to the main health domains investigated, including metabolic, gastrointestinal, musculoskeletal, reproductive, inflammatory, neurological, and psychological outcomes. Patterns of benefit, consistency of findings, and methodological limitations were examined across included studies. The synthesis focused on identifying clinically relevant evidence supporting medicinal plant-based naturopathy as a complementary therapeutic approach. No pooled meta-analysis was performed unless sufficient homogeneity in study design and outcome measures was present.

Results

Search Results

**Figure 1:**
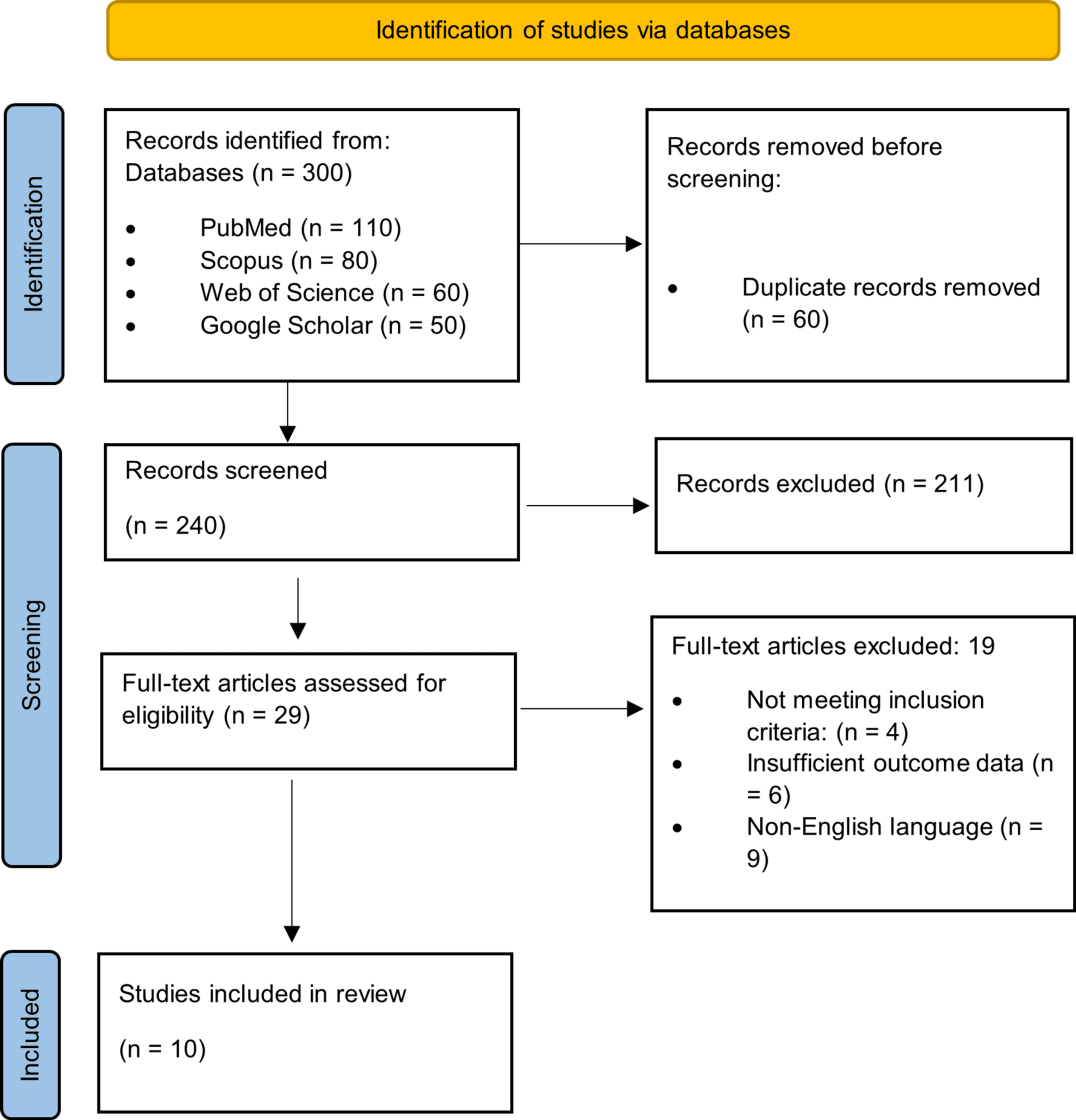
PRISMA flow diagram illustrating the study selection process

A thorough literature search was carried out across the selected electronic databases to identify relevant studies examining the therapeutic potential of medicinal plant-based naturopathy. The search identified a total of 300 records from PubMed (n = 110), Scopus (n = 80), Web of Science (n = 60), and Google Scholar (n = 50). After removal of duplicate records (n = 60), 240 records were screened by title and abstract for relevance to the objectives of this review in accordance with the PRISMA 2020 framework [26]. Of these, 211 records were excluded because they were not directly related to medicinal plant-based therapeutic interventions or did not involve relevant clinical or life science evidence. A total of 29 full-text articles were assessed for eligibility against the predefined inclusion and exclusion criteria. During full-text assessment, 19 articles were excluded, including articles not meeting the inclusion criteria (n = 4), articles with insufficient outcome data (n = 6), and non-English-language articles (n = 9). Following systematic screening and eligibility assessment, 10 studies were included in the final review. The complete process of study identification, duplicate removal, screening, eligibility assessment, exclusion with reasons, and final inclusion is presented in the PRISMA flow diagram (Figure 1) [26].

Study Characteristics

These investigations examined a wide range of botanical interventions, including peppermint (*M. piperita*) oil, Tongxie Yaofang herbal formulation, and silymarin from *S. marianum*. Other included interventions were mulberry leaf extract (*M. alba*), *B. serrata*, *W. somnifera* root extract, and *O. tenuiflorum* extract [28,29,30,31,32]. The included studies involved populations with pre-hypertension or stage 1 hypertension, diarrhoea-predominant irritable bowel syndrome, and irritable bowel syndrome with functional dyspepsia [30,31,33]. Other study populations included patients with non-alcoholic steatohepatitis, adults with moderate spondylitis, women with menopausal symptoms, men with mild sexual dysfunction, adults with psychological stress, and healthy normoglycaemic adults [28,29,32,34]. Intervention durations varied across studies, ranging from short-term supplementation trials of 20 to 30 days to eight-week interventions, a 56-day intervention, a 28-day intervention, and a longer 48-week trial [28,35]. Sample sizes varied depending on study design and target population, and most trials included adult participants receiving a controlled intervention with a placebo comparator [36,37]. Evidence from the included studies supported the potential role of plant-derived compounds in improving metabolic health, gastrointestinal symptoms, and musculoskeletal symptoms [32,33,37]. Additional evidence supported possible roles in hepatic outcomes, hormonal balance, reproductive health, and psychological well-being [28,29,36]. These findings indicate growing clinical interest in medicinal plants as complementary strategies within naturopathic medicine [30,34,37]. The characteristics of the included studies are shown in Table 1. The PICO framework derived from the included clinical studies is presented in the Appendix.

**Table 1:** Characteristics of the included clinical studies IBS-D: diarrhoea-predominant irritable bowel syndrome; NAS: NAFLD activity score; BASDAI: Bath Ankylosing Spondylitis Disease Activity Index; NDI: Neck Disability Index; IIEF: International Index of Erectile Function; TCM: traditional Chinese medicine; TXYF: Tongxie Yaofang; RCT: randomised controlled trial

Study	Medicinal plant / extract	Sample size	Condition	Interventions	Primary outcomes / key findings
Vani et al., [28]	*Withania somnifera *(ashwagandha root extract)	60 women	Women experiencing menopausal symptoms	Ashwagandha extract 300 mg daily for 56 days	Significant reduction in Menopause Rating Scale scores along with improvements in hormonal balance and quality-of-life measures.
Khanna et al., 2026 [29]	*Withania somnifera* (ashwagandha root extract)	76 men	Healthy adult men with mild sexual dysfunction	300 mg extract twice daily for eight weeks	Significant improvements in sexual desire, IIEF scores, sperm parameters, and overall sexual satisfaction without major adverse events.
Sinclair et al., [30]	*Mentha piperita *(peppermint oil)	40 participants	Adults with pre-hypertension or stage-1 hypertension	Peppermint oil 100 µL/day compared with placebo for 20 days	Study protocol designed to evaluate changes in systolic blood pressure, cardiometabolic indicators, psychological status, and sleep-related parameters.
Liang et al., [31]	Tongxie Yaofang (TCM formula: *Atractylodes macrocephala*, *Paeonia lactiflora*, *Citrus aurantium*,* Saposhnikovia divaricata*)	96 participants	Adults with diarrhoea-predominant irritable bowel syndrome (IBS-D)	TXYF granules 3.7 g twice daily for eight weeks	Symptom improvement was observed in both TXYF and placebo groups, with no statistically significant difference in IBS-SSS response rates.
Mamatha et al., [32]	*Boswellia serrata* extract ± curcumin	105 participants (94 completed)	Adults with moderate spondylitis	400 mg/day *Boswellia* extract or *Boswellia*–curcumin complex for 28 days	Marked reductions in pain, stiffness, BASDAI, and NDI scores; the combination formulation demonstrated stronger anti-inflammatory effects.
Ivashkin et al., [33]	Menthol + limonene + gingerol supplement	56 participants	Patients diagnosed with IBS or IBS with functional dyspepsia	Supplement capsule (730 mg/day) combined with standard therapy for 30 days	Significant reduction in symptom severity scores compared with placebo, with no serious adverse effects reported.
Lopresti et al., [34]	*Ocimum tenuiflorum* (holy basil) extract	Approximately 100 adults	Adults experiencing psychological stress	Holy basil extract (Holixer™) compared with placebo for eight weeks	Significant decreases in perceived stress along with improvements in mood and sleep quality relative to placebo.
Navarro et al., [35]	*Silybum marianum* (silymarin)	78 randomized	Patients with non-alcoholic steatohepatitis (NASH) without cirrhosis	Silymarin 420 mg or 700 mg compared with placebo three times daily for approximately 48 weeks	Histological NAS score did not show significant improvement compared with placebo; treatment was well tolerated and demonstrated a favourable safety profile.
Solhi et al., [36]	*Silybum marianum* / silymarin	64 patients completed the trial; 33 in the silymarin group and 31 in the placebo group	Patients with non-alcoholic steatohepatitis (NASH) confirmed by abdominal sonography and persistently elevated AST/ALT levels	Silymarin 210 mg/day orally for eight weeks, given as 70 mg three times daily, compared with placebo; both groups also received lifestyle advice, including a low-fat, low-carbohydrate diet, exercise, and weight-loss guidance	Silymarin produced a greater reduction in hepatic enzymes than placebo. ALT decreased from 91.3 ± 21.3 to 38.4 ± 11.8 IU/L in the silymarin group, compared with 84.6 ± 23.3 to 52.3 ± 29.9 IU/L in controls; AST also decreased significantly. The authors concluded that silymarin may be useful as a complementary treatment for NASH.
Lown et al., [37]	*Morus alba *(mulberry leaf extract)	37 participants	Healthy normoglycemic adults	Mulberry leaf extract administered with a maltodextrin test meal	Significant reductions in postprandial blood glucose and insulin responses compared with placebo.

Risk-of-Bias Assessment

According to the Cochrane Risk of Bias framework, the quality of the methodological approach used in the identified studies was assessed using the ROBviz tool [27]. Most of the incorporated studies were well designed in terms of methodology, especially in terms of randomisation, and placebo-controlled trials have been used. Many studies were sufficiently detailed in their description of allocation concealment and random sequence generation to reduce the likelihood of selection bias. Most of the studies employed the blinding of participants, investigators, and outcome assessors to minimise the chances of performance and detection bias. Since participant withdrawal and follow-up outcome were highly documented, the bias of attrition was generally considered insignificant. Some studies were considered to have a risk of reporting bias because of incomplete information on protocol registration or rather selective publication of findings. Most of the research was of moderate to high quality in general, based on the risk of bias assessment, and the findings of the systematic review have a reasonable likelihood that they could be backed by clinical evidence. The methodological quality and risk of bias of the included studies were assessed by the Cochrane Risk of Bias framework; the results are presented in Table 2.

**Table 2:** Risk-of-bias assessment of the included studies using the Cochrane Risk of Bias

Study (author, year)	Randomization process	Allocation concealment	Blinding of participants and personnel	Blinding of outcome assessment	Incomplete outcome data	Selective reporting	Overall risk of bias
Vani et al., [28]	Low	Low	Low	Low	Low	Low	Low
Khanna et al., 2026 [29]	Low	Low	Low	Low	Low	Low	Low
Sinclair et al., [30]	Low	Low	Low	Low	Low	Moderate	Low
Liang et al., [31]	Low	Low	Low	Low	Low	Low	Low
Mamatha et al., [32]	Low	Low	Low	Low	Low	Moderate	Low
Ivashkin et al., [33]	Low	Low	Low	Low	Low	Low	Low
Lopresti et al., [34]	Low	Low	Low	Low	Low	Low	Low
Navarro et al., [35]	Low	Low	Low	Low	Low	Moderate	Low
Solhi et al., [36]	Low	Low	Low	Low	Low	Moderate	Low
Lown et al., [37]	Low	Low	Low	Low	Low	Low	Low

**Figure 2:**
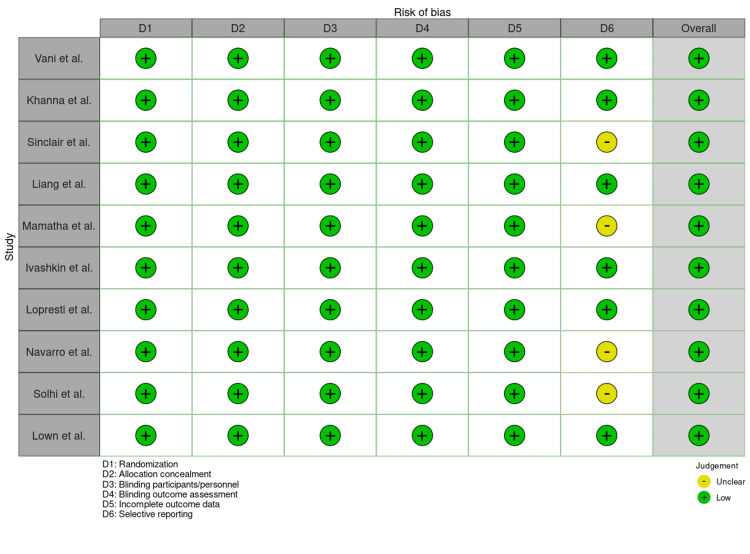
ROBvis traffic-light plot showing the risk-of-bias assessment D1: randomization; D2: allocation concealment; D3: blinding of participants/personnel; D4: blinding of outcome assessment; D5: incomplete outcome data; D6: selective reporting. Yellow circles indicate studies judged as having moderate risk/some concerns in the selective reporting domain.

The results were visualised using a ROBvis traffic-light plot (Figure 2).

Therapeutic Effects of Medicinal Plant Interventions on Physiological Disorders

Several studies included in this review addressed the role of medicinal plants in physiological disorders and were structured according to the affected system. Several of these plants also have a history of traditional use; for example, peppermint has been traditionally used for digestive discomfort, Tongxie Yaofang in traditional Chinese medicine for bowel-related symptoms, *S. marianum* for liver-related conditions, and mulberry leaf in traditional medicine for metabolic support. In cardiovascular disorders, peppermint oil supplementation was examined among individuals with pre-hypertension or stage 1 hypertension, with outcomes including systolic blood pressure, cardiometabolic indicators, psychological status, and sleep-related parameters [30]. In gastrointestinal disorders, conventional herbal preparations such as Tongxie Yaofang have been researched for diarrhoea-predominant irritable bowel syndrome, where symptom improvement was observed in both intervention and placebo groups, although no statistically significant difference was found in IBS-SSS (Irritable Bowel Syndrome Symptom Severity Score) response rates (79.2% vs. 70.8%; p = 0.348) [31]. A supplement containing menthol, limonene, and gingerol was also evaluated in gastrointestinal disorders and showed potential benefits in reducing symptom severity among patients with irritable bowel syndrome or irritable bowel syndrome with functional dyspepsia (p = 0.009) [33].

**Figure 3:**
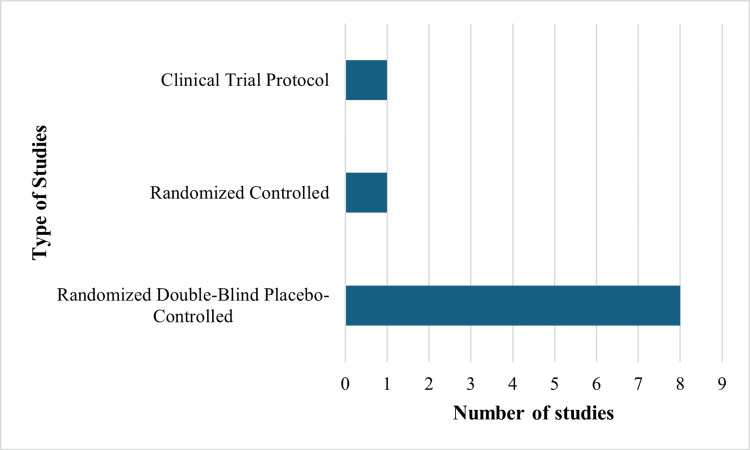
Therapeutic effects of medicinal plant interventions

In hepatobiliary disorders, silymarin from* S. marianum* was assessed for hepatoprotective effects in patients with non-alcoholic steatohepatitis. One trial reported that silymarin was safe and well tolerated, although histological improvement was not significantly different from placebo (p = 0.79) [35]. Another trial reported greater reductions in hepatic enzymes, including alanine transaminase (ALT) and aspartate transaminase (AST), in the silymarin group compared with placebo [36]. In metabolic disorders, mulberry leaf extract showed beneficial effects, especially in reducing postprandial blood glucose and insulin responses after a maltodextrin test meal (glucose response reduced by 14.0-22.0%; insulin response reduced by 23.8-24.7%) [37]. The likely mechanisms include smooth muscle relaxation and modulation of gut motility for peppermint-derived compounds, anti-inflammatory and gut-regulatory effects for Tongxie Yaofang, antioxidant and anti-inflammatory hepatoprotection for silymarin, and inhibition of carbohydrate digestion and absorption for mulberry leaf extract. Collectively, these studies point to the potential of plant-derived compounds to affect cardiovascular parameters, gastrointestinal symptoms, hepatobiliary outcomes, and metabolic pathways [33,36,37]. The therapeutic effects of medicinal plant interventions on physiological and metabolic disorders are shown in Figure 3.

Therapeutic Effects of Medicinal Plant Interventions on Musculoskeletal, Reproductive, and Psychological Health

**Figure 4:**
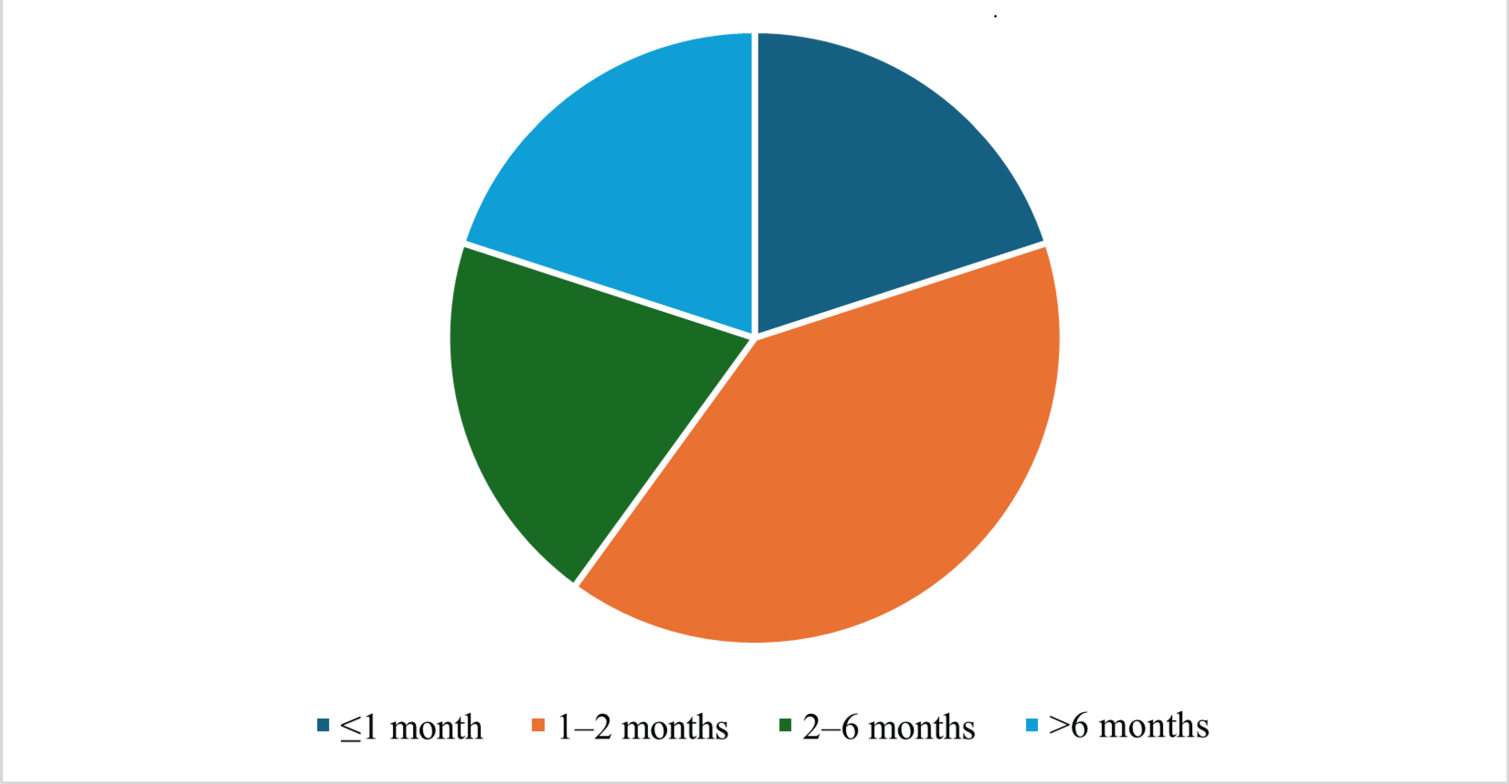
Therapeutic effects of the duration of medicinal plant interventions

In addition to physiological disorders, several studies evaluated the effects of medicinal plants on musculoskeletal, reproductive, menopausal, and psychological health outcomes. Ashwagandha (*W. somnifera*) was tested in placebo-controlled clinical trials involving women with menopausal symptoms and men with mild sexual dysfunction [28,29]. Compared with placebo, these studies reported improvements in hormonal balance, quality-of-life indicators, reproductive function, sexual desire, International Index of Erectile Function (IIEF) scores, sperm parameters, and overall sexual satisfaction [28,29]. *B. serrata *extract, alone or in combination with curcumin, was evaluated against a placebo in adults with moderate spondylitis and was associated with reductions in pain, stiffness, Bath Ankylosing Spondylitis Disease Activity Index (BASDAI) scores, and Neck Disability Index (NDI) scores [32]. Another placebo-controlled study investigated the effects of *O. tenuiflorum* extract in adults with psychological stress and reported favourable changes in perceived stress, mood, and sleep quality compared with placebo [34]. Together, these results show that medicinal plants may have a wide range of therapeutic potential beyond metabolic and physiological disorders. These botanical interventions, through their adaptogenic, anti-inflammatory, or neuroprotective properties, may contribute to improving well-being and supporting the integration of naturopathic approaches within modern healthcare systems. The therapeutic effects of medicinal plant interventions on musculoskeletal, reproductive, and psychological health outcomes are shown in Figure 4.

Discussion

The findings of this review suggest that medicinal plant-based interventions may provide complementary therapeutic benefits across selected physiological, musculoskeletal, reproductive, psychological, gastrointestinal, and hepatometabolic conditions. However, the strength of evidence varies across interventions, and the results should be interpreted cautiously because of differences in study design, sample size, intervention formulation, dosage, duration, and outcome measures. The strongest supportive findings were observed in reproductive, menopausal, and psychological outcomes. Ashwagandha (*W. somnifera*) was associated with improvements in menopausal symptoms, hormonal balance, quality-of-life measures, sexual function, sperm parameters, and overall sexual satisfaction [38]. Similarly, *O. tenuiflorum*, commonly known as holy basil, showed beneficial effects on stress, mood, and sleep in adults with stress-related symptoms [39], suggesting possible adaptogenic effects that may support stress regulation, mood, sleep quality, and overall well-being. These findings indicate that adaptogenic plants may be useful in stress-related and reproductive health contexts, although larger trials with standardised extracts are still required.

Anti-inflammatory and analgesic effects were mainly relevant to musculoskeletal and chronic inflammatory conditions. Medicinal plants have been studied for arthritis and related musculoskeletal disorders because several plant-derived compounds may reduce inflammatory signalling, oxidative stress, and pain-related pathways [40]. These effects support their possible role as adjunctive options rather than replacements for standard care, particularly in chronic conditions where long-term symptom control and tolerability are important. Cardiovascular and metabolic findings were also clinically relevant. Specific medicinal plants, including *Allium sativum*, *Crataegus monogyna*, *Hibiscus sabdariffa*, *Camellia sinensis*, and *Olea europaea*, have been studied for their potential roles in the management of cardiovascular diseases. These plants contain relevant phytochemicals, including organosulfur compounds from *Allium sativum*, flavonoids and procyanidins from *Crataegus monogyna*, anthocyanins from *Hibiscus sabdariffa*, catechins from *Camellia sinensis*, and phenolic compounds such as oleuropein and hydroxytyrosol from *Olea europaea* [41]. These phytochemicals may influence cardiovascular risk factors through antioxidant, anti-inflammatory, lipid-modulating, and vascular-protective effects. However, the clinical relevance of these mechanisms depends on formulation quality, bioavailability, dosage, treatment duration, and comparison with standard therapies.

Gastrointestinal evidence showed mixed but useful findings. Tongxie Yaofang was compared with a placebo in diarrhoea-predominant irritable bowel syndrome and showed symptom improvement in both groups without a statistically significant between-group difference. In contrast, a supplement containing menthol, limonene, and gingerol used with standard therapy produced greater symptom reduction than placebo plus standard therapy in patients with irritable bowel syndrome or functional dyspepsia [31,33]. These results suggest that some plant-derived combinations may improve gastrointestinal symptoms, but efficacy is not uniform across all formulations.

The hepatometabolic findings were also mixed. Silymarin showed a favourable safety profile in non-alcoholic steatohepatitis, although one trial did not show significant histological improvement compared with placebo, while another reported greater reductions in hepatic enzymes. Mulberry leaf extract showed beneficial effects on postprandial glucose and insulin responses. These findings suggest that plant-derived compounds may influence hepatic and metabolic pathways, but stronger clinical evidence is needed before definitive therapeutic claims can be made.

Beyond clinical efficacy, sustainability and biodiversity remain important considerations. Cultivation-related threats, including diseases caused by root-knot nematodes, may reduce the yield and availability of medicinal plant resources, requiring effective agricultural and conservation strategies [42]. Ethnobotanical research continues to document traditional medicinal knowledge and may support the discovery of new therapeutic compounds [43]. Modern genomic and biotechnological approaches, including next-generation sequencing, can further support conservation, authentication, and sustainable use of medicinal plant biodiversity [44]. The evidence also indicates that medicinal plant applications extend beyond human clinical use. Plant-based products have been evaluated as topical options in veterinary dermatology, suggesting broader biological relevance across species [45]. Traditional medical systems have also described neuroprotective herbal remedies that may influence oxidative stress, inflammation, and neuronal degeneration in neurodegenerative disorders [46,47]. However, these areas require more rigorous translational and clinical studies before firm conclusions can be drawn.

The results of this review support the potential of medicinal plants as complementary therapeutic agents, but the evidence remains heterogeneous. The most consistent clinical relevance was observed where trials used defined formulations, placebo or standard-care comparators, measurable outcomes, and safety monitoring. Future studies should prioritise larger randomised controlled trials, standardised plant extracts, clear active-compound profiling, clinically meaningful endpoints, long-term safety assessment, and direct comparison with standard treatment where appropriate. Such evidence is necessary to determine which medicinal plant-based interventions can be integrated into evidence-based naturopathic and complementary healthcare practice.

Limitations and Future Directions

This review has certain limitations that should be recognised. The number of clinical studies included was limited, and the sample size of several of the trials was relatively small. Differences in the design of studies and the duration and dosage of plant extracts used also made direct comparison between studies difficult. In addition, most interventions were concerned with short-term effects, and this limits our understanding of the long-term efficacy and safety of medicinal plant-based therapies.

Future research should be directed to the performance of larger and well-designed randomised controlled trials using standardised methodologies. Further studies are also needed for investigating the medicinal plant interventions in terms of pharmacological mechanisms, optimal dosages and long-term safety. The synthesis of traditional medicinal knowledge and modern scientific thinking processes may be useful to support the strengthening of the evidence base and development of effective plant-based therapeutic strategies.

## Conclusions

Medicinal plant-based naturopathy is gaining respect as a form of therapy, as a complementary therapy in the prevention and treatment of various degrees of health conditions. The findings presented in the research incorporated in this systematic review highlight the possibility of plant-derived compounds in enhancing physiological and psychological health effects. Clinical evidence has demonstrated the beneficial effects of several medicinal plants, including *W. somnifera*, *O. tenuiflorum*, *B. serrata*, *M. alba*, and *M. piperita*, in the management of several conditions, such as metabolic disorders, gastrointestinal diseases, musculoskeletal pain, reproductive health issues, and stress-related conditions. Plants are mainly attributed to their bioactive phytochemicals with antioxidant activity, reduced inflammation activity, adaptive activities, and their metabolic regulatory properties. These mechanisms are involved in the enhanced function of physiology and may be able to reduce the risk or severity of certain chronic diseases. In all, Medicinal plants constitute a significant resource in the development of a natural therapeutic strategy. Continued scientific investigation through well-designed clinical trials, as well as pharmacological studies, will be needed to further validate their efficacy, safety, and mechanism of action and add credibility to their role in evidence-based naturopathic medicine.

## Appendices

Table 3 shows the PICO framework for the clinical studies included in this systematic review. It summarises the population, intervention, comparator, and outcome elements used to evaluate medicinal plant-based naturopathic interventions.

**Table 3:** PICO framework derived from the included clinical studies ALT: alanine aminotransferase; AST: aspartate aminotransferase; BASDAI: Bath Ankylosing Spondylitis Disease Activity Index; IBS: irritable bowel syndrome; IBS-SSS: Irritable Bowel Syndrome Severity Scoring System; IIEF: International Index of Erectile Function; NAFLD: non-alcoholic fatty liver disease; NDI: Neck Disability Index; PICO: Population, Intervention, Comparator, Outcome

Included study	Population	Intervention	Comparator	Outcomes assessed
Vani et al. [28]	Women experiencing menopausal symptoms	Ashwagandha root extract, 300 mg daily for 56 days	Placebo	Menopause Rating Scale score, hormonal balance, quality-of-life measures, safety outcomes
Khanna et al. [29]	Healthy adult men with mild sexual dysfunction	Ashwagandha root extract, 300 mg twice daily for eight weeks	Placebo	Sexual desire, International Index of Erectile Function scores, sperm parameters, sexual satisfaction, safety outcomes
Sinclair et al. [30]	Adults with pre-hypertension or stage 1 hypertension	Peppermint oil, 100 µL/day for 20 days	Placebo	Systolic blood pressure, cardiometabolic indicators, psychological status, sleep-related parameters
Liang et al. [31]	Adults with diarrhoea-predominant irritable bowel syndrome	Tongxie Yaofang granules, 3.7 g twice daily for eight weeks	Placebo	IBS symptom severity, IBS-SSS response rate, gastrointestinal symptom improvement, safety outcomes
Mamatha et al. [32]	Adults with moderate spondylitis-associated pain and stiffness	Boswellia serrata extract or Boswellia serrata–curcumin co-delivery formulation, 400 mg/day for 28 days	Placebo	Pain, stiffness, Bath Ankylosing Spondylitis Disease Activity Index, Neck Disability Index, inflammatory response
Ivashkin et al. [33]	Patients with irritable bowel syndrome or irritable bowel syndrome with functional dyspepsia	Supplement containing standardized menthol, limonene, and gingerol, 730 mg/day for 30 days, with standard therapy	Placebo with standard therapy	IBS symptom severity, abdominal symptoms, functional dyspepsia symptoms, safety outcomes
Lopresti et al. [34]	Adults experiencing psychological stress	Standardized Ocimum tenuiflorum extract for eight weeks	Placebo	Perceived stress, mood, sleep quality, stress-related outcomes, safety outcomes
Navarro et al. [35]	Non-cirrhotic patients with non-alcoholic steatohepatitis	Silymarin 420 mg or 700 mg three times daily for approximately 48 weeks	Placebo	Histological NAFLD Activity Score, liver-related outcomes, tolerability, safety profile
Solhi et al. [36]	Patients with non-alcoholic steatohepatitis confirmed by abdominal sonography and persistently elevated AST/ALT levels	Silymarin 210 mg/day orally for eight weeks with lifestyle advice	Placebo with lifestyle advice	Alanine aminotransferase, aspartate aminotransferase, hepatic enzyme reduction, treatment response
Lown et al. [37]	Healthy normoglycaemic adults	Mulberry leaf extract administered with a maltodextrin test meal	Placebo with maltodextrin test meal	Postprandial blood glucose response, insulin response, glucose tolerance, gastrointestinal tolerability
